# Combinatorial Therapeutic Potential of Stem Cells and Benzimidazol Derivatives for the Reduction of Liver Fibrosis

**DOI:** 10.3390/ph16020306

**Published:** 2023-02-15

**Authors:** Maryam Iqbal, Sulaiman Shams, Huma Rafiq, Momin Khan, Shahid Khan, Umer Sadique Khattak, Sahib Gul Afridi, Fehmida Bibi, Angham Abdulrhman Abdulkareem, Muhammad Imran Naseer

**Affiliations:** 1 Department of Biochemistry, Abdul Wali Khan University Mardan, Mardan 23200, Khyber Pakhtunkhwa, Pakistan; 2 Department of Chemistry, Abdul Wali Khan University Mardan, Mardan 23200, Khyber Pakhtunkhwa, Pakistan; 3 College of Veterinary Sciences, The University of Agriculture, Peshawar 25130, Khyber Pakhtunkhwa, Pakistan; 4 Special Infectious Agents Unit, King Fahd Medical Research Centre, King Abdulaziz University, Jeddah 21589, Saudi Arabia; 5 Department of Medical Laboratory Technology, Faculty of Applied Medical Sciences, King Abdulaziz University, Jeddah 21589, Saudi Arabia; 6 Department of Biochemistry, Faculty of Science, King Abdulaziz University, Jeddah 21589, Saudi Arabia; 7 Center of Excellence in Genomic Medicine Research, King Abdulaziz University, Jeddah 21589, Saudi Arabia

**Keywords:** stem cells, benzimidazol, compound, hepatocytes, rat

## Abstract

**(1) Background:** Liver fibrosis is currently one of the top ten causes of death worldwide. Stem cells transplantation using mesenchymal stem cells (MSCs) is an alternative therapy which is used in the place of organ transplant, due to the incapacity of stem cells to endure oxidative stress in the damage site, thus affecting the healing process. The present study aimed to enhance the therapeutic potential of MSCs using combined therapy, along with the novel synthetic compounds of benzimidazol derivatives. **(2) Methods:** Eighteen compound series (benzimidazol derivatives) were screened against liver fibrosis using an in vitro CCl_4_-induced injury model on cultured hepatocytes. IC50 values were calculated on the bases of LDH assay and cell viability assay. **(3) Results:** Among the eighteen compounds, compounds (**10**), (**14**) and (**18**) were selected on the basis of IC50 value, and compound (**10**) was the most potent and had the lowest IC50 value in the LDH assay (8.399 ± 0.23 uM) and cell viability assay (4.73 ± 0.37 uM). Next, these compounds were combined with MSCs using an in vitro hepatocytes injury culture and in vivo rat fibrotic model. The effect of the MSCs + compounds treatment on injured hepatocytes was evaluated using LDH assay, cell viability assay, GSH assay and real-time PCR analysis and immuno-staining for caspase-3. Significant reductions in LDH level, caspase-3 and apoptotic marker genes were noted in MSCs + compounds-treated injured hepatocytes. In vivo data also showed the increased homing of the MSCs, along with compounds after transplantation. Real-time PCR analysis and TUNEL assay results also support our study. **(4) Conclusions:** It was concluded that compounds (**10**), (**14**) and (**18**) can be used in combination with MSCs to reduce liver fibrosis.

## 1. Introduction

The liver serves as the digestive organ of all vertebrates; it detoxifies metabolites, synthesizes proteins and produces the biochemicals needed for both digestion and development [1]. Almost all medicines are recognized by the human body as foreign substances (xenobiotics), which then undergo a number of chemical reactions (metabolism) to make them suitable for elimination. Portal veins, which carry drugs and xenobiotics in a nearly undiluted form, supply the liver with around 75% of its blood directly from the digestive system and the spleen. However, the liver’s crucial role in chemical clearance and transformation makes it susceptible to drug-induced damage. The liver can be harmed by more than 100 drugs [2].

Liver fibrosis is a condition caused by chronic inflammation or damage in parenchyma cells. Various diseases and medicines can repeatedly or continuously harm the liver, resulting in fibrosis. Scars are formed in the liver during liver fibrosis. The build-up of extracellular matrix components that are rich in fibrillar collagens and the lack of matrix turnover are characteristics of a fibrogenic response. The seventh to tenth most common cause of death globally is liver fibrosis [3]. The most frequent causes of liver damage in the United States are alcohol addiction, viral hepatitis and non-alcoholic fatty liver disease, often known as non-alcoholic steato-hepatitis [4]. The effective treatment of chronic liver damage might result in fibrosis regression, due to the cessation of the fibro-genic reaction after the clearance of hepatic myo-fibroblasts and restoration of the fibro-lytic pathways [5]. The anti-fibrotic and anti-inflammatory drugs for the treatment of liver fibrosis that are currently on the market are ineffective and sometimes cause major side effects or harm.

Whole-organ allograft transplantation is now the gold standard for treating end-stage liver disease. Due to the limited availability of suitable organs and high expense and challenging surgical procedures involved, the use of liver transplantation is limited [6]. Stem cell-based therapy as an alternative has gained interest due to the promising results of preclinical and clinical trials [7]. Numerous scientific and clinical studies have demonstrated that cell-based therapies using MSCs have favorable effects on liver fibrosis [8]. MSCs are delivered by the portal vein or teil vain [9], intra-hepatic or spleen [10]. MSCs are the most often employed stem cells in cell therapy and tissue engineering [11]. MSCs stimulate tissue regeneration and aid in healing when they are triggered via certain procedures or when they are injected directly into the site of an injury [12]. MSCs have the ability to travel from their niche into the peripheral circulation and through vessel walls to reach target tissues [11]. MSCs can be differentiated into hepatic cells and have the capacity to restore liver functioning [13,14,15]. Poor cell survival after transplantation is one of the key factors determining the therapeutic efficiency of stem cell treatment. This is caused, in part, by the fact that transplanted stem cells cannot survive oxidative or inflammatory stresses at the site of an injury [16]. Therefore, the development of alternate treatment regimens is urgently needed. To counteract these effects, various types of pre-treatment methods have begun to be investigated. It was reported that the pre-treatment of cells with the antioxidant called N-acetylcysteine (NAC) significantly enhanced the survival of muscle-derived stem cells (MDSCs) and improved heart function when transplanted to a model of myocardial infarction [17]. Melatonin is used as an antioxidant, according to a new study of the use of adipose-derived mesenchymal stem cells (ADSC) to improve the potential of the cells for combatting acute interstitial cystitis in rats [18]. It has also been found that edaravone can significantly affect the capacity of hUC MSCs to repair hepatic tissue by raising their antioxidant levels. Hepatic functions were improved and the host liver was regenerated [16]. MSCs treated with selenium reduced liver fibrosis in a CCl_4_ mice model [19]. MSCs combined with vitamin E can reduce liver fibrosis in CCl_4_-treated injured mice [20]. It has been reported that MSCs and melatonin showed great therapeutic effects on a CCl_4_-induced liver fibrosis model [21]. Benzimidazol is a family of various compounds with a wide range of biological activities, including antiviral, anticancer, antihypertensive, antifungal, anthelmintic, anti-inflammatory and antioxidant properties. Furthermore, a small number of N-substituted benzimidazole derivatives have been demonstrated to have potent antiviral action against HIV, hepatitis C virus, influenza virus (IV), herpes simplex virus-1 (HSV-1), picorna and human cytomegalovirus [22]. It is found in natural cyanocobalamine, as well as in synthetic medicines such as omeprazole, mebendazole and acetamidazole [23]. Albendazole belongs to the benzimidazole family, and is an anthelminthic drug with a broad spectrum of activities [24]. MSCs in combination with albendazole had a therapeutic impact on brain inflammation, gliosis and apoptosis, as well as showing a substantial reduction in brain damage biomarkers. Following ABZ treatment, MSC transplantation can restore wounded liver tissue in experimentally infected rats without completely removing the hydatid cyst [25]. In light of the abovementioned literature, the study in this article was designed with the aim to evaluate—for the first time—the therapeutic potentials of novel benzimidazol derivatives against liver fibrosis and their combined therapeutic potential—along with MSCs—on damaged hepatocytes induced by CCl_4_ in in vitro hepatocytes and also in an experimental rat model.

## 2. Results

### 2.1. In Vitro Studies

#### 2.1.1. Culturing and Characterization of Rat Hepatocytes

Hepatocytes from male Wister rat liver were isolated using the Ferrigno (2010) two-step perfusion method [26], and hepatocytes viability was checked by trypan blue assay, which showed that 85–95% of hepatocytes were viable. The Ferrigno (2010) method was then used to isolate hepatocytes from male Wister rat liver and trypan blue assay were conducted to check the viability of hepatocytes, which show that 85–95% of the hepatocytes were viable. The morphology of the cells was noted under a phase-contrast microscope after 2 h and 24 h of culturing cells in an RPMI medium. After 2 h, the cells were binucleated in a spherical shape, similar to the morphology of normal hepatocytes. The cells were flattened and polygonal in shape after 24 h of culturing, as shown in Figure S1. After culturing, hepatocytes were characterized at both the RNA and protein levels, using real-time PCR and immunochemistry for the hepatocytes-specific marker. Cultured cells exhibited hepatocyte-specific markers, i.e., albumin, CK-8 and CK-18 according to real-time PCR analysis. As an endogenous control, β actin was employed (Figure S2A). Hepatocytes’ ability to store glycogen is also crucial; hence, PAS-staining was used to assess the functional status of the cultured hepatocytes. After PAS-staining, the cells became purple, indicating that the cultured hepatocytes were also functionally active (Figure S2B). To examine the protein expression of certain hepatic cell markers in the cultured cells, immunostaining was performed. DAPI was used to label the nuclei of the cells. Albumin, CK-8, CK-18 and AFP-positive cells were found after immunostaining (Figure S2Ca–d).

#### 2.1.2. Hepatocytes Injury Analysis

For the preparation of the CCl_4_-treated hepatocytes injury culture, rat hepatocytes cells were treated with 5mM of CCl_4_ concentrations for 6 h [27]. LDH assay (Figure S3A), trypan blue assay (Figure S3B), GSH assay (Figure S3C) and real-time PCR analysis (Figure S3D) were used to examine the hepatic marker expression (CK-8 and albumin), anti-apoptotic (Bcl-xL) markers and apoptotic (*BAX* and caspase-3) markers showed the maximum level of CCl_4_-induced hepatocytes injury.

#### 2.1.3. In Vitro Screening of Compounds

The novel compounds of benzimidazol derivatives 1–18 were screened against liver fibrosis [28]. In vitro CCl_4_-induced injury culture was used for screening. LDH assay and trypan blue assay were performed to find the viability of the cells (Figure S4A,B), from which the IC50 values of the compounds were calculated. GSH/GSSH assay was also performed to calculate the total glutathione level (Figure S4C). Among all of the compounds, compound (**10**) was found to be the most potent compound because it has the lowest IC50 value and the highest GSH/GSSG value. In addition to compound (**10**), other compounds found to be potent were (**14**) and (**18**). Therefore, these three compounds were selected for in vitro and in vivo study in combination with MSCs. Compound structures and names, along with the IC50 values, are shown in Table 1.

#### 2.1.4. In Vitro Enhanced Hepatocytes Survival after Treatment with MSCs + Compounds

**Figure 1:**
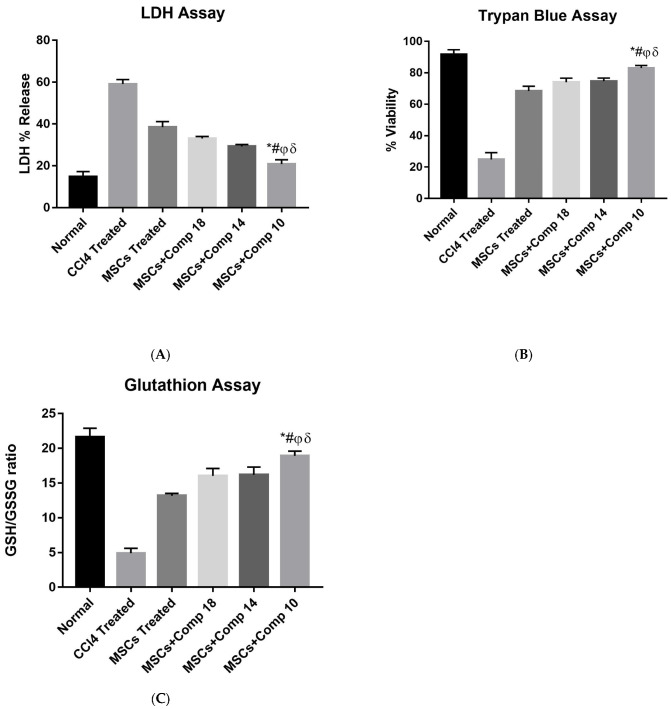
In vitro enhanced hepatocytes survival after treatment with MSCs + compounds: (**A**) LDH assay of hepatocytes co-cultured with MSCs + compounds. % LDH release was significantly decreased in MSCs + compound (**10**)-treated hepatocytes, compared to CCl_4_, only MSCs-treated hepatocytes and MSCs + compounds (**18**)- and (**14**)-treated hepatocytes, as shown by bars (*). (**B**) Cell viability assay of hepatocytes co-cultured with MSCs + compounds. Cell viability was highly increased in MSCs + compound (**10**)-treated hepatocytes, compared to CCl_4_-treated, only MSCs-treated hepatocytes and + MSCs + compound (**18**)- and (**14**)-treated hepatocytes, as shown by bars with alphabet letter (*). (**C**) Glutathione assay of hepatocytes co-cultured with MSCs + compounds. GSH level was significantly increased in MSCs + compound (**10**)-treated hepatocytes, compared to CCl_4_-treated, only MSCs-treated hepatocytes and MSCs + compounds (Co Cultured)- and (**14**)-treated hepatocytes, as shown by bars with symbols *. Mean ± SEM (n = 3). Bars with symbols show significance with each other. * *p* < 0.02 for MSCs+Comp10 vs. CCl4; # *p* < 0.05 for MSCs + Comp10 vs. MSCs; φ *p* < 0.05 for MSCs + Comp10 vs. MSCs+Comp18; δ *p* < 0.05 for MSCs + Comp10 vs. MSCs + Comp14.

First, the hepatocytes were exposed to a CCl_4_ dose (5 mM for 6 h) followed by treatment with compounds (**10**), (**14**) and (**18**) for 24 h. After the compounds treatment, MSCs were co-cultured with hepatocytes in a Transwell system with and without compounds. The co-cultures of hepatocytes were divided into nine groups, i.e., normal, CCl_4_-treated, three groups of only compound-treated, MSCs-treated and three groups of MSCs + compounds-treated. A significant decrease in the level of LDH was seen in compounds-treated and MSCs + compounds-treated injured hepatocytes (Figure 1A). However, treatment with MSCs + compounds, predominantly compound (**10**), showed a significantly higher decrease in cell injury than any other treatment group. Cell viability (Figure 1B) and GSH level (Figure 1C) were highly increased in MSCs + compounds (**10**)-treated hepatocytes.

#### 2.1.5. Gene Expression Analysis of Co-Cultured hepatocytes model

**Figure 2:**
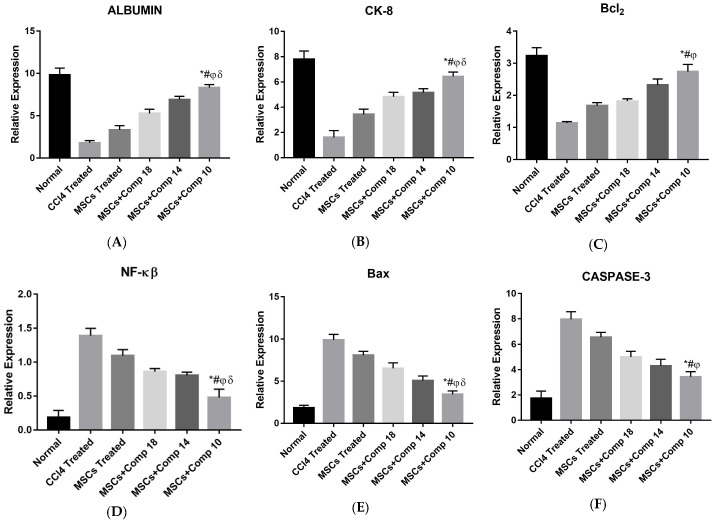
Real-time PCR analysis of in vitro hepatocytes co-cultured with MSCs + compounds. Expression of normal hepatic genes (albumin (**A**) and CK-8 (**B**)) and anti-apoptotic (*BCl_2_* (**C**)) markers genes was increased and apoptotic (*BAX* (**E**), caspase-3 (**F**) and *NF-κB* (**D**)) genes decreased in MSCs + compounds-treated hepatocytes, especially in the case of + MSCs + compound (**10**)-treated hepatocytes, as shown by bars with symbols *. Mean ± SEM (n = 3). Bars with symbols show significance with each other. * *p* < 0.02 for MSCs + Comp10 vs. CCl4; # *p* < 0.05 for MSCs + Comp10 vs. MSCs; φ *p* < 0.05 for MSCs + Comp10 vs. MSCs+Comp18; δ *p* < 0.05 for MSCs + Comp10 vs. MSCs + Comp14.

Gene expression analysis showed reductions in apoptotic markers such as *BAX*, caspase-3 and *NF-κB* in hepatocytes treated with MSCs and compounds alone, as compared to being CCl_4_-treated, but a significant reduction was observed after treatment with MSCs + compounds, particularly compound (**10**), compared to all other groups. Likewise, an increased level of anti-apoptotic markers *BCl_2_* was observed after treatment by MSCs or compounds, but a greater increase was observed after the combined treatment of MSCs + compounds (compound (**10**)), compared to CCl_4_-treated hepatocytes (Figure 2). 

#### 2.1.6. Immunostaining for Protein Expression of Caspase-3 in In Vitro Co-Culture Model

**Figure 3:**
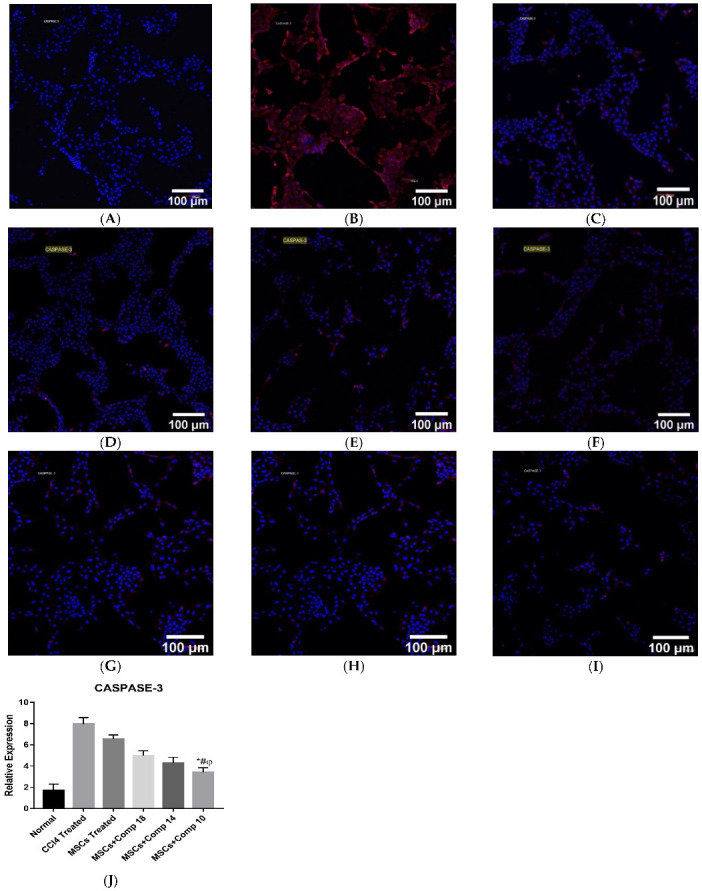
Immunostaining for the detection of Caspase-3 in in vitro co-cultured hepatocytes model: (**A**) normal, (**B**) CCl_4_-treated, (**C**) compound (**18**), (**D**) compound (**14**), (**E**) compound (**10**)-treated hepatocytes, (**F**) MSCs-treated, (**G**) MSCs + compound (**18**), (**H**) MSCs + compound (**14**), (**I**) MSCs + compound (**10**), (**J**) graphical representation of fluorescence intensity of caspase-3 in all groups. Expression of caspase-3 protein was significantly decreased in MSCs + compound (**10**)-treated hepatocytes, compared to all other groups, shown by bar with symbols *. Mean ± SEM (n = 3). Bars with symbols show significance with each other. * *p* < 0.02 for MSCs + Comp10 vs. CCl_4_; # *p* < 0.05 for MSCs + Comp10 vs. MSCs; φ *p* < 0.05 for MSCs + Comp10 vs. MSCs + Comp18 & 14.

Immunostaining was performed for the detection of caspase-3 in an in vitro co-culture model. The primary antibody used was rabbit polyclonal IgG (1:500, abcam), and the secondary antibody used was goat anti-rabbit IgG (1:1000, abcam). The MSCs + compounds-treated group showed low Casepase-3 protein expression, compared to the MSCs-, only compound- and CCl_4_-treated groups (Figure 3A–I).

### 2.2. In Vivo Studies

#### 2.2.1. Comparative Morphological Study of Liver

**Figure 4:**
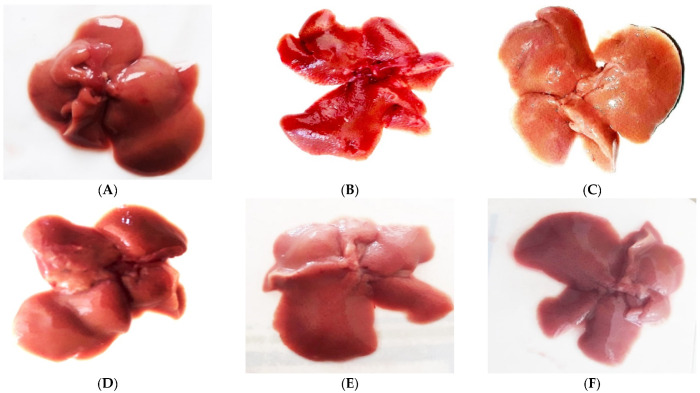
Liver morphology after MSCs transplantation along with compounds: (**A**) normal, (**B**) CCl_4_-treated, (**C**) MSCs-treated, (**D**) MSCs + compounds (**18**), (**E**) MSCs + compounds (**14**), (**F**) MSCs + compounds (**10**). Liver morphology showed that fibrosis was greatly reduced in MSCs + compound (**10**)-treated experimental model, with less scars and a radish color, resembling with normal liver.

All experimental animals were anesthetized; the livers were isolated and the morphology of livers was observed. The comparative morphology can be seen in Figure 4A–F. The morphology of CCl_4_-treated animal liver was more brown in color, with large scars (Figure 4B). The MSCs-treated animal liver was less brown and scars were decreased (Figure 4C). The scars gradually decreased in MSCs + compounds-treated groups (Figure 4D–F), but significant decreases in scars were noted in the MSCs + compound (**10**)-treated group, and the color of liver was radish, close to the normal liver morphology (Figure 4F). 

#### 2.2.2. Homing of Transplanted MSCs in Fibrotic Liver

**Figure 5:**
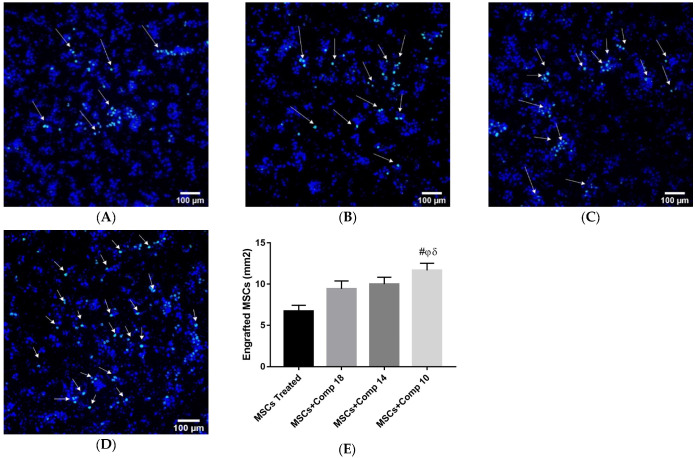
MSCs homing in injured liver 15 days post-transplantation: (**A**) injured liver with MSCs transplantation; (**B**) injured liver subjected to combined treatment MSCs + compound (**18**); (**C**) MSCs + compounds (**14**); (**D**) MSCs + compounds (**10**); (**E**) engrafted cells quantification in different experimental models. (100×; scale bar: 100 μM). Bars along with different symbols show significance with each other at *p* < 0.05. # *p* < 0.05 for MSCs + Comp10 vs. MSCs; φ *p* < 0.05 for MSCs + Comp10 vs. MSCs + Comp18; δ *p* < 0.05 for MSCs + Comp10 vs. MSCs + Comp14.

The MSCs were labeled with PKH67 (green cell-linker dye) and, for the examination of nuclei DAPI, were transplanted into the liver of a fibrotic rat model along with compounds or in the absence of compounds for the detection of the homing of MSCs. The improved homing in fibrotic liver of the models subjected to MSCs + compounds (Figure 5A–D) treatment was examined, as shown by the high number of PKH-67/DAPI co-labelled cells, compared to MSCs, that were only transplanted in the liver, thus showing an increase in the homing and engraftment of MSCs in fibrotic livers treated with MSCs along with compound (**10**).

#### 2.2.3. MSCs + Compounds Transplantation Effects on Apoptosis

**Figure 6:**
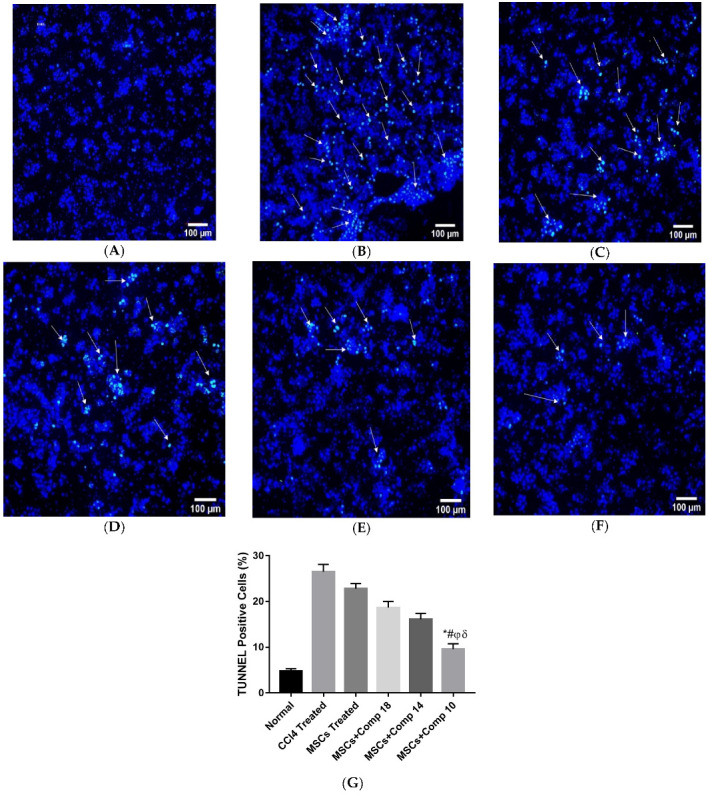
Assessment of apoptosis in experimental models using the TUNEL apoptotic. Assay in all experimental models (**A**–**F**). TUNEL apoptotic cells are shown by positive nuclei (green) in liver slices: (**A**) normal, (**B**) CCl_4_-treated, (**C**) MSCs-treated, (**D**) MSCs + compound (**18**)-treated, (**E**) MSCs + compound (**14**)-treated, (**F**) MSCs + compounds (**10**)-treated, (**G**) graphical representation of TUNEL-positive cells. Among all treated animal models, MSC + compound (**10**)-treated rat model showed the most reduced apoptotic cells, as shown by bars with symbols *. Mean ± SEM (n = 3). Bars with symbols show significance with each other. * *p* < 0.02 for MSCs + Comp10 vs. CCl_4_; # *p* < 0.05 for MSCs + Comp10 vs. MSCs; φ *p* < 0.05 for MSCs + Comp10 vs. MSCs + Comp18; δ *p* < 0.05 for MSCs + Comp10 vs. MSCs + Comp18. To stain the nuclei (Blue) DAPI was used (20×; scale bar: 100 µm).

The amount of TUNEL-positive cells in the fibrotic liver considerably decreased in MSCs + compounds (**18**), (**14**) and (**10**)-transplanted groups (Figure 6), when compared with CCl_4_; with only MSCs-treated groups, the number of tunnel-positive cells significantly decreased in MSCs + compounds (**18**, **14** and **10**)-treated experimental models. Among all groups, the + MSCs + compounds (**10**)-transplanted group showed a highly decreased number of apoptotic cells (Figure 6F).

#### 2.2.4. Analysis for Gene Expression after MSCs + Compounds Transplantation

**Figure 7:**
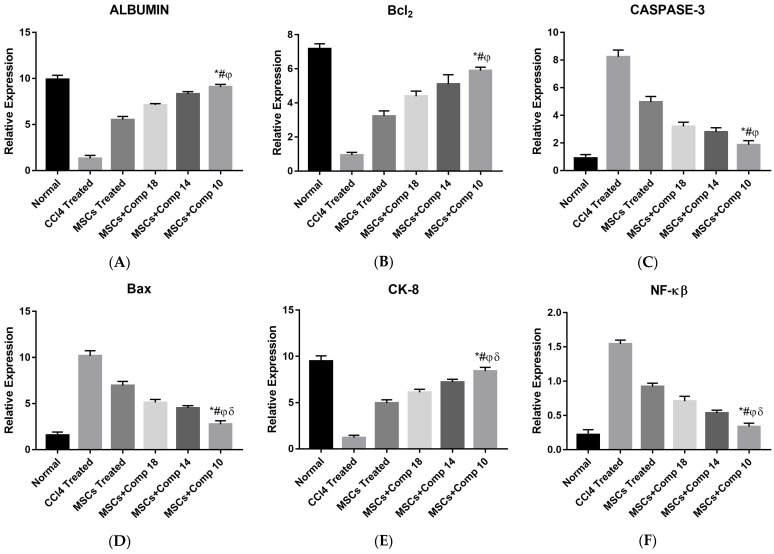
Real-time PCR analysis liver tissue after MSCs + compounds transplantation. Hepatic marker genes (albumin (**A**), CK-8 (**E**)) and anti-apoptotic (*Bcl-xI* and *BCl_2_* (**B**)) were increased and apoptotic (*BAX* (**D**), caspase-3 (**C**), NF-kβ (**F**) and TNF-α) genes were decreased 2 weeks after transplantation. *p* < 0.05 for MSCs + compounds-treated rats, compared to CCl_4_-treated and only MSCs-treated animal models. The MSCs + compound (**10**)-treated model showed highly expressed hepatic and anti-apoptotic genes and the reduced apoptotic gene was most potent, as compared to the other treated animal model, as shown by bars with different symbols. * *p* < 0.02 for MSCs + Comp10 vs. CCl_4_; # *p* < 0.05 for MSCs + Comp10 vs. MSCs; φ *p* < 0.05 for MSCs + Comp10 vs. MSCs + Comp18; δ *p* < 0.05 for MSCs + Comp10 vs. MSCs + Comp18.

Real-time PCR analysis was performed for all experimental models; the expression of the hepatic, anti-apoptotic and apoptotic genes was studied after 2 weeks of MSCs transplantation. In rats treated with MSCs + compound (**10**), the expression level of apoptotic (caspase-3, TNF-α, NF-kβ and *BAX*) genes greatly decreased, and the expression of the liver marker genes (albumin and CK-8) and anti-apoptotic (Bcl-xI and *BCl_2_*) genes was up-regulated, compared to CCl_4_-treated and only MSCs-transplanted rat models (Figure 7).

#### 2.2.5. Improved Liver Function after MSCs + Compounds Treatment

**Figure 8:**
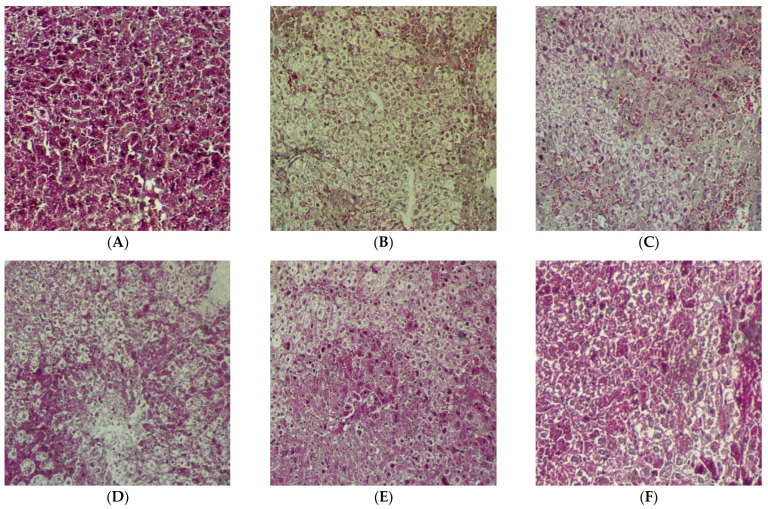
PAS-staining for glycogen storage in the liver sections of all experimental models: (**A**) normal, (**B**) CCl_4_-treated, (**C**) MSCs-treated, (**D**) + MSCs + compound (**18**)-treated, (**E**) MSCs + compound (**14**)-treated (**F**) MSCs + compound (**10**)-treated. MSCs + compound (**10**) show increased recovery of glycogen storage (20×; scale bar: 100 µm).

Glycogen storage is a key function of hepatocytes. In all experimental groups, PAS-staining was used to assess glycogen storage levels. When compared to MSC alone, MSC + compounds (**18**), (**14**) and (**10**) demonstrated improved glycogen storage recovery. The MSC + compound (**10**)-treated group showed highly increased recovery of glycogen storage (Figure 8F).

#### 2.2.6. Biochemical Functions

**Figure 9:**
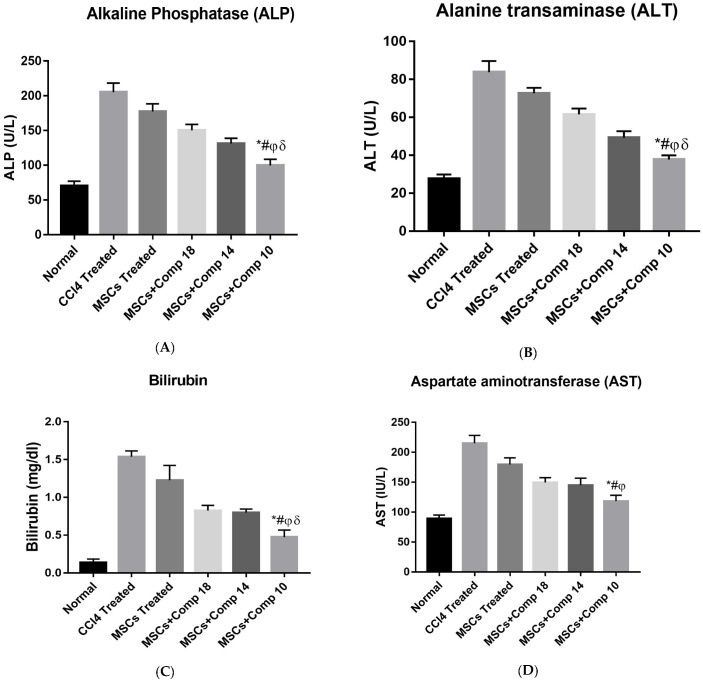
Liver function tests after MSCs + compounds transplantation: (**A**) alkaline phosphatase; (**B**) alanine transaminase; (**C**) bilirubin; (**D**) aspartate aminotransferase levels in all experimental models after 2 weeks of transplantation. *p* value < 0.05 was considered significant. MSCs + compounds (**10**)-transplanted animals show a greatly reduced enzymes level, shown by bars with the symbol (*). * *p* < 0.05 for MSCs+Comp10 vs. CCl_4_; # *p* < 0.05 for MSCs + Comp10 vs. MSCs; φ *p* < 0.05 for MSCs + Comp10 vs. MSCs+Comp18; δ *p* < 0.05 for MSCs + Comp10 vs. MSCs + Comp18.

After two weeks of MSCs transplantation, serum, AST, ALT, ALP and bilirubin levels were compared between the seven experimental rat models to measure liver function. Compared to other animal groups, MSCs + compounds (**18**)-, (**14**)- and (**10**)-implanted rat models had considerably lower levels of enzymes. Among these, MSCs + compound (**10**)-treated animals showed a highly decrease level of enzymes, compared to the others, as shown in (Figure 9).

#### 2.2.7. Histopathological Analysis

**Figure 10:**
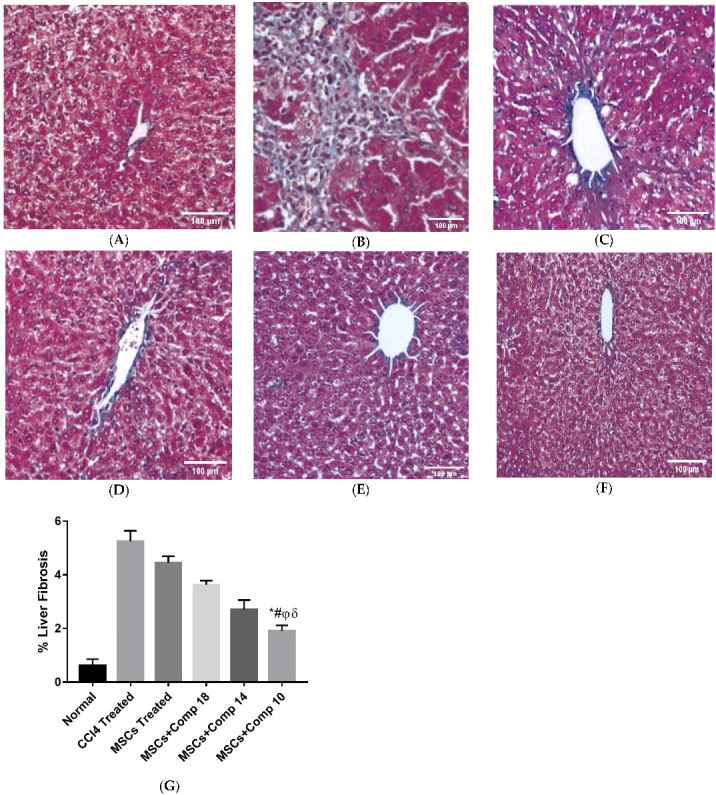
Masson trichome-staining for collagen (blue) in liver tissue after MSCs + compounds treatment: (**A**) normal; (**B**) CCl_4_-treated; (**C**) MSCs-treated; (**D**) MSCs + compound (**18**); (**E**) MSCs + compound (**14**); (**F**) MSCs + compound (**10**); (**G**) bar graph showing % of liver fibrosis in different groups (100×; scale bar: 100 μM). Bars and different symbols show significance with each other at *p* < 0.05. Collagen amount greatly decreased in the MSCs + compound (**10**)-treated rat experimental model compared to all other groups, as shown by bars with symbols. * *p* < 0.05 for MSCs + Comp10 vs. CCl4; # *p* < 0.05 for MSCs+Comp10 vs. MSCs; φ *p* < 0.05 for MSCs + Comp10 vs. MSCs + Comp18; δ *p* < 0.05 for MSCs + Comp10 vs. MSCs + Comp18.

In rats transplanted with MSCs and MSCs + compounds, liver histology was examined using Masson trichrome staining. Normal liver sections were found to have a structure which lacks fibrosis and inflammation (Figure 10A), whereas the CCl_4_-treated model showed fibrosis by the loss of structural integrity and a high amount of deposited collagen (Figure 10B). Compared to the CCl_4_-treated liver (Figure 10B) and only MSCs-transplanted liver (Figure 10C), the transplantation of MSCs + compounds dramatically reduced CCl_4_-induced fibrosis and collagen levels (Figure 10D–F). For the quantification of data, Image J software was used. The scale was first briefly changed to micrometers, and was then converted to greyscale. Then, the blue-stained collagen was segmented (isolated) using thresholding and the threshold area was measured. 

## 3. Discussion

Liver illness is responsible for over two million fatalities each year globally, with one million of these deaths attributable to cirrhosis complications. Fibrosis of the liver is currently one of the top ten causes of death worldwide [29]. The final phase of liver fibrosis is cirrhosis, during which the architecture of the liver is disrupted and the extracellular matrix is deposited [30]. Whole-organ allograft transplantation is currently an available treatment of last-stage liver diseases. However, due to the paucity of suitable organs, high pricing and surgical challenges, the use of liver transplantation is restricted [6]. The use of MSCs in cell-based therapy, on the other hand, has been shown to be effective in curing liver fibrosis in numerous basic and clinical research works [8]. One of the most important aspects affecting the healing effectiveness of stem cell treatments is the poor survival of cells after transplantation. This is partially due to the transplanted stem cells’ incapacity to endure the oxidative stress and inflammatory responses present in the damage site [16]. Increasing the differentiation potential of MSCs is urgently needed in order to convert them into hepatic cells and repair the potential of MSCs to repair the injured tissue or organ by combination therapy. 

In our study, MSCs (MSCs) combined with a novel synthetic compound of benzimidazol derivatives treatments were investigated for their capacity to reduce liver fibrosis in experimental models. These compounds were first screened against fibrosis using an in vitro hepatocytes culture, in which these compounds also increased in glutathione (GSH) level, which shows the antioxidant activity of these derivatives. These compounds were first screened in vitro on CCl_4_-induced injured hepatocytes, in which these compounds also increased in glutathione (GSH) level, which shows the antioxidant activity of these derivatives. An in vitro CCl_4_-induced injury culture of rat hepatocytes was prepared for the screening of its compounds and to determine the therapeutic effect of the combination of MSCs and new chemical entities in regard to decreasing liver fibrosis.

Among the eighteen compounds, three compounds were selected for combination therapy with MSCs, on the basis of low IC50 values in LDH assay, trypan blue exclusion assay and increased glutathione level (Figure S4). Compounds (**10**), (**14**) and (**18**) were active, in comparison to the other compounds of the series. Compound (**10**) was highly potent, compared to the other compounds. It has previously been reported that glutathione treatment can improve liver functioning [31]. Our results agree with this because the glutathione level increased significantly in our models. Compound (**10**) is a benzimidazole derivative consisting of a methyl group (-CH3) attached to a benzene ring at position 3, while all the other compounds in this series do not have this group; thus, the high potency of this compound may be due to the presence of this methyl group. In small-molecule medicines, the methyl group is one of the most prevalent carbon fragments. This simplest alkyl group may change both the biological and physical aspects of the molecule and was found in more than 67 percent of the top-selling medications in 2011. 

The simple modification of C-H to C-Me boosts a drug candidate’s IC50 value by more than 100-fold [32]. Another compound, (**14**), of the benzimidazol series, consists of three methoxy groups (O-CH3). In pharmacology, the methoxy group, when attached to benzene ring, takes an important and often vital pharmacophoric part. The benzene ring and the methoxy group, during metabolic oxidation, can both produce hydroxy groups. These hydroxy groups increase the solubility of water and the removal of the metabolite, which results in the termination of the activity of the drug due to the pharmacokinetic impact [33]. The methoxy group may increase or decrease the activity of a drug. In this research, due to the presence of methoxy groups, the compound showed a high level of activity. Another compound, (**18**), also consists of one methoxy group, which showed a high level of activity compared to the other compounds of their series, but a low level of activity compared to compounds (**10**) and (**14**). These three compounds were then studied in combination with MSCs using CCl_4_-induced injury both in vitro and in vivo. The injured hepatocytes were co-cultured with these three compounds, with only MSCs and with MSCs + compounds. MSCs + compounds reduced injury, causing a smaller amount LDH release and a higher number of viable cells in this treatment group (Figure 1A,B).

The real-time PCR analysis of the combined treatment—MSCs + compounds—co-cultured model showed a considerable decrease in pro-apoptotic and an increase in anti-apoptotic and hepatic gene expressions (Figure 2), compared to the other experimental models. Here, it is reported that a significant decrease in pro-apoptotic and increase in anti-apoptotic and hepatic marker expressions improved liver functioning in the CCl_4_-treated rat model [21]. The MSCs + + compound (**10**) showed a significant decrease in the amount of LDH released, increased cell viability and an increased level of GSH/GSSG (Figure 2A–C). The MSCs + compounds treatment of the CCl_4_-induced injury reduced caspase-3 protein expression in a co-culture model when studied by immunohistochemistry (Figure 3). Protein expression is highly reduced in MSCs + + compound (**10**)-treated models (Figure 3I). Caspase-3 suppression is connected with reduced hepatic cell damage and death [34].

The valuable influence of the combined MSCs and compound treatment on the survival of hepatocytes in vitro was extended to in vivo to show that these compounds can improve the hepatic milieu and can also increase hepatocytes survival here, resulting in the greater effectiveness of MSCs therapy in fibrotic liver repair. Compounds were injected and then MSCs were transplanted in the liver of a fibrotic model induced by CCI_4_, in vivo. MSCs combined with the compounds increased the homing of MSCs in the injured liver of a CCl_4_-treated rat model. In the present study, it was demonstrated that the transplanted MSCs combined with compounds were considerably better at homing into the injured liver of rat and did not die away after a further 2 weeks of CCI_4_ treatment, in comparison to only MSCs-treated animals (Figure 5). 

The results of this study coincide with the results obtained by the increasing antioxidant levels in hUCMSCs, with edaravone being shown to have a big impact on their ability to heal hepatic tissue [16]. Functional analysis demonstrated that MSCs + compounds-treated groups showed considerably decreased serum, ALT, AST, ALP and bilirubin levels in in vivo (Figure 9).

The results obtained in this study show that the restoration of the amount of ALP and bilirubin in serum enable better liver functions [35]. The results of PAS-staining confirmed that the decrease in glycogen level was restored in livers transplanted with MSCs + compounds, compared to CCl_4_-treated and only MSCs-treated animals (Figure 8).

In the present study, real-time PCR analysis (Figure 7) confirmed that the MSCs + compounds treatment of a CCl_4_-treated injured liver fibrotic model considerably decreased apoptotic (*BAX*, caspase-3, *NF-Kβ* and TNF-α) genes expression, and up-regulated anti-apoptotic (*BCl_2_* and Bcl-xL) marker genes. The low expression of apoptotic and high expression of anti-apoptotic markers in the liver may be due to the enhanced therapeutic potential of MSCs combined with compounds. These results coincide with the experiments, which proved that the up-regulation of anti-apoptotic gene Bcl-xL and *BCl_2_* expression and the down-regulation of *BAX*, caspase-3, *NF-κB* and TNF-α are indicative of improved liver functioning [36]. It was also reported that increased antioxidant levels in hUCMSCs with edaravone can have a big impact on their ability to heal hepatic tissue. As shown in the literature, hepatic functions were improved and the host liver was regenerated [16].

## 4. Materials and Methods

### 4.1. Isolation and Culturing of Rats Hepatocytes

Hepatocytes were isolated from male Wister rats using a modified in situ collagenase perfusion method [26]. The cell viability of the hepatocytes cell, which was isolated from rats, was measured through a trypan blue exclusion test. Then, the hepatocytes cells were isolated; cultured in collagen coated plates in an RPMI medium; augmented with 100 Units/mL of penicillin, 100 µg/mL of streptomycin, 50 ng/mL of EGF and 10% FBS; and then incubated at 37 °C in a 5% CO_2_ atmosphere. The morphology of the isolated cells was examined after 2 h and 24 h of culturing, under a phase-contrast light microscope, for the confirmation of their hepatic origin. RNA was extracted from the cultured cells using an E.Z.N.A.^®^RNA Isolation Kit procedure, and the cDNA was synthesized using a cDNA Synthesis Kit. One µI of cDNA generated from the cultured hepatocytes was used for the RT-PCR analysis of specific markers (CK-8, CK-18 and albumin) of hepatocytes. Primer size and sequences are given in Table 1. As an internal control, *β-Actin* was used. Immuno-cyto chemistry was used for cultured cells to determine specific markers (albumin, CK-18 and CK-8) and their expression of hepatocytes at a protein level. A PAS-staining test was performed for glycogen storage to examine whether the cultured cells were functionally active hepatocytes.

### 4.2. In Vitro CCl_4_-Induced Injury of Hepatocytes

In vitro CCl_4_-induced injury to hepatocytes was given by treating the cultured hepatocytes with a 5 mM concentration of CCl_4_ in DMSO, as described previously [27]. Non-treated cultured hepatocytes were considered normal. After 6 h of injury with CCl_4_, the media from the cultured cells were collected and stored for the LDH assay.

### 4.3. Selection and Screening of Compounds

Eighteen compounds of benzimidazol derivatives [28] were selected and screened against liver fibrosis using in vitro CCl_4_-induced injury to cultured hepatocytes. Different concentrations of compounds (1.5 µm, 3.1 µm, 6.25 µm, 12.5 µm, 25 µm, 50 µm, and 100 µm) were prepared for screening, in which three compounds showed excellent activity.

### 4.4. Lactate Dehydrogenase (LDH) and Cell Viability Assay

The hepatocytes medium was centrifuged at 250× *g* for 4 min, and the LDH activity of the supernatant was evaluated at 492 nm, according to the kit protocol (Roche). The cell viability assay for CCl_4_-treated injured cells, compound-treated, MSCs-treated and + MSCs + compounds-treated hepatocytes cells was performed using a trypan blue-exclusive methodology. The number of viable cells was counted by dividing the number of trypan blue negative cells by the total number of cells observed and multiplying this by 100.

### 4.5. GSH/GSSH Assay

The glutathione assay was performed for the CCl_4_-treated injured cells, compound-treated, MSCs-treated and MSCs + compounds-treated hepatocytes cells, according to the kit’s procedure (GSH/GSSG-Glo™ Assay kit, promega); briefly, the medium containing the test compounds was removed from cells. Both the lysis reagent were prepared and added into each well; then, the plates were shacked, and 50 µL of luciferin generation reagent was added to each well, shook for 30 min, and then, 100 µL of luciferin detection reagent was added to each well. The plates were shook again; after waiting for 15 min, the luminescence was measured using a Tecan Microplate Reader (Tecan Group Ltd., Männedorf, Zürich, Switzerland).

### 4.6. Immunostaining

Immunostaining was conducted to find the expression of caspase-3 protein in normal hepatocytes, CCl_4_-treated and compounds-treated, MSCs-treated and MSCs + compounds-treated hepatocyte. First, the cells were washed 3 times with PBS, each for 5 min, fixed in 4% formaldehyde for 30 min at room temperature and permeabilized with 0.1% triton for 10 min; then, nonspecific binding was inhibited by blocking with 2% BSA in PBS for 45 min. The cells were then incubated with primary antibodies for 4 h at room temperature. The primary antibody used for caspase-3 was rabbit monoclonal (1:1000, abcam) and the secondary antibody was goat anti-rabbit (1:500, abcam, Cambridge, UK). The samples were incubated for 45 min at room temperature (RT), and then were washed with PBS and incubated with DAPI for 2–5 min at room temperature. Then, the samples were again washed with PBS, and subsequently mounted with vecta sheets. The samples were then observed using a Fluoview FV 3000 microscope (Olympus, Tokyo, Japan) and pictures were taken.

### 4.7. MSCs Isolation and Culturing

MSCs were isolated from the femur bone and tibia bone of Wister rats that were 250 g–300 g in weight according to the protocol previously described [36]. The cells were cultured in a 25 cm^2^ culture flask in IMDM medium, which was supplemented with 20% fatal bovine serum (FBS), 100 µg/mL of streptomycin and 100 U/mL of penicillin, and incubated at a temperature of 37 °C in an atmosphere containing 5% CO_2_. On the third day, the culture medium was changed to plated cells. For the confirmation of the existence of the MSCs and, in order to remove hematopoietic stem cells, FACS analysis was performed. CD90, CD44, CD105 and CD34 antibodies were used.

### 4.8. In Vitro Injured Hepatocytes Co-Culture Model

In vitro injury was given for 6 h by treating cultured hepatocytes with a 5 mM concentration of CCl_4_ in DMSO, as described previously [27]. The co-cultured model was established by culturing injured hepatocytes treated with MSCs and compounds (**18**), (**14**) and (**10**) for 24 h in a Transwell culture system with DMEM (sigma) medium containing 10% FBS, 100 μg/mL of streptomycin and 100 U/mL of penicillin. The co-culture was undertaken using a porous Transwell membrane, with a pore size of 0.4 mm (BD Biosciences). Hepatocytes were first seeded at a density of 1.5 × 10^5^/cm^2^ on the collagen-coated 6-well plate. Once attached, the MSCs were seeded onto the Transwell membrane inserts at a density of 1.5 × 10^4^. The hepatocytes were divided into six groups: normal, CCl_4_-treated injured, CCl_4_-treated injured co-cultured with MSCs alone, CCl_4_-treated injured co-cultured combined with MSCs + compound (**18**), CCl_4_-treated injured co-cultured combined with MSCs + compound (Luciferin Generation Reagent) and CCl_4_-treated injured co-cultured combined with MSCs + compound (**10**). After 24 h of co-culturing, the treated hepatocytes cells were then collected for the extraction of their RNA, LDH cytotoxicity assay, trypan blue assay, glutathione assay and immunostaining.

### 4.9. Gene Expression Analysis

From the CCl_4_-treated injured cells and other treated cells, and also from liver tissues samples, RNA was extracted using an E.Z.N.A. ^®^ Total RNA kit, according to the kit protocol. The quantification of RNA was conducted using a Spectrophotomter Nano drop; ND-1000 and cDNA were synthesized by a BIO-RAD I Script^tm^cDNA synthesis kit using RT-PCR.

Real-time PCR analysis was carried out with SYBR Green PCR Super Mix (Bio-Rad, Hercules, CA, USA) and 2 µI of cDNA. The procedure of PCR consisted of an initial period of denaturation at a temperature of 94 °C for 4 min, 35 cycles of denaturation for 45 s and then annealing at 56 °C–58 °C for 45 s and an extension at 72 °C for 45 s, followed by a final step of extension at 72 °C for 10 min. The expression levels of albumin, cyto-keratin 8, *Bcl_2_*, *BAX*, Bcl-xL, TNF-α, NF-κβ and caspase-3 were measured. The comparative CT method (∆∆Ct value) was used to measure the relative expression of target genes. As a reference, gene β-actin was used. All primer sequences are given in Table 2.

### 4.10. CCl_4-_Induced LiverFibrotic Model

Male Wister rats were used for the liver fibrotic model. Rats were kept with free access to water and food in sterilized cages. For the preparation of the model, 1 µL/g of body weight of CCl_4_ (1:1 in olive oil) was injected intraperitoneally for four weeks, twice per week, as described previously [27].

### 4.11. In Vivo Compounds Treatment

After the completion of 4 weeks of CCl_4_ treatment, compounds (**18**), (**14**) and (**10**) were injected at a dose of 50 μg/kg intraperitoneally. The rats were divided into seven groups: normal, CCl_4_, MSCs and two groups of MSCs + compounds-treated rats; each experimental group consisted of six animals.

### 4.12. MSCs Transplantation

Cultured MSCs were trypsinized and then labelled with PKH-67 (green) cell linker fluorescent dye (Sigma-Aldrich, St. Louis, MO, USA), as described previously [37]. MSCs were transplanted to MSCs + compounds groups for four hours after injecting compounds. All groups were euthanized, and the abdomen was cut to expose the liver. Approximately 10^7^ cells were transplanted in 1 mL of PBS into a liver lateral and median lobes using a 30 G syringe.

### 4.13. Periodic Acid Schiff (PAS) Assay 

After 15 days of the MSCs and compounds treatment, PAS-staining was performed for all experimental groups for the measurement of glycogen storage levels in liver sections. First, the 5 µm thick microtome sections of the liver were de-paraffinized. Tissue sections were then incubated for 5 min in periodic acid at RT. Then, tissue sections were washed with distilled H_2_O and stained with Schiffs reagent for 15 min and then hematoxylin-stained for 90 s. Tissue sections were then washed with tap water; the sections were then mounted and observed under microscope.

### 4.14. Masson Trichrome-Staining

The formalin-fixed liver tissue was dehydrated and then embedded in paraffin. The tissue was cut into 5 µm thick sections by a microtome Leica RM 2155 (Leica Biosystems, Wetzlar, Germany) and slides were prepared. Then, they were stained for the collagen using a Masson trichome staining kit (Abcam), according to the kit protocol. Collagens were observed under an Olympus BX-61 microscope in the fibrotic liver and pictures were captured with a Digital Camera camera (Olympus digital camera DP70, Olympus Optical Co., Ltd., Tokyo, Japan). Using Image J software, the quantification of collagen percentage was conducted regarding the stained liver sections [27]. 

### 4.15. Biochemical Analysis

Blood samples were collected and centrifuged for each experimental group at 8000 rpm for 15 min in order to isolate the serum. Bilirubin, alanine transaminase (ALT), alkaline phosphatase (ALP) and aspartate transaminase (AST) testing is a common group of tests used to check liver health. High levels of ALT, ALP, AST and bilirubin release in blood may be a sign of a liver injury or disease. ALT, ALP, bilirubin and AST levels were measured in serum, according to kit (Centronics, Wartenberg, Germany) protocol.

### 4.16. TUNELAssay for Hepatic Apoptosis

The TUNEL assay, an appropriate tissue processing technique, is a relatively fast, reproducible and quantitative method for detecting apoptosis in tissue. The TUNEL assay was performed using a TUNEL Apoptosis Detection Kit (Merck-millipore, Temecula, CA, USA) for the detection of apoptosis in each group. The 5 µm thick liver sections in paraffin were used in the TUNEL assay. Three sections were selected for each rat. An Olympus BX-61 microscope was used for the examination of the tissues of the experimental groups, and images were taken with a Digital Camera DP-70 (Olympus, Japan). The number of apoptotic hepatocytes was calculated in each section.

### 4.17. Statistical Analysis

All data are presented as mean ± SD. The analysis of fibrosis percentage between groups was performed using one-way ANOVA. The graphs were made with the help of Graph Pad prism 7. *p* value < 0.05 was considered significant.

## 5. Conclusions

In the present study, it is demonstrated that, based on the IC50 values found, compounds (**10**), (**14**) and (**18**) were active against liver fibrosis. These three compounds also increased the glutathione (GSH/GSSG) level in an in vitro experimental model, and can be used as an antioxidant. Furthermore, these compounds increased the potential of MSCs for liver fibrosis reduction. In conclusion, compound (**10**) was the most potent among all the compounds, can be used as a lead compound for the synthesis of drugs against liver fibrosis, and the potential of the use of MSCs against liver fibrosis is enhanced using these compounds ((**10**), (**14**) and (**18**)) combined with MSCs. MSCs combined with compound (**10**) significantly improved the liver functions. Here, we report a different therapeutic approach, combining synthetic compounds with MSCs for the treatment of liver fibrosis.

## Figures and Tables

**Table 1:** Compound structures and formulas with IC50 values.

S. No.	Structure of Compound	IC50 (µM) ± SD	
		% LDH Release	% Viability
1	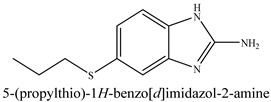	34.33 ± 0.1	9.66 ± 0.67
2	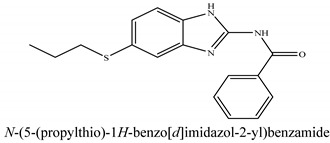	13.54 ± 0.13	14.3 ± 0.21
3	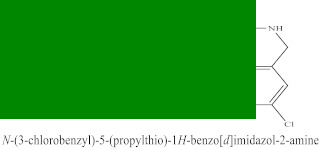	11.17 ± 0.54	17.86 ± 0.42
4	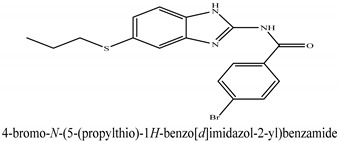	23.26 ± 0.32	11.78 ± 0.15
5	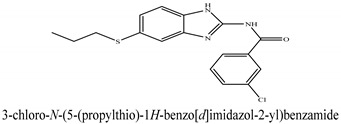	17.22 ± 0.86	13.55 ± 0.75
6	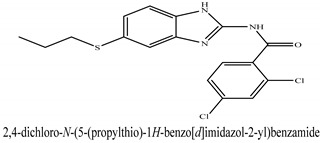	51.8 ± 0.72	8.27 ± 0.26
7	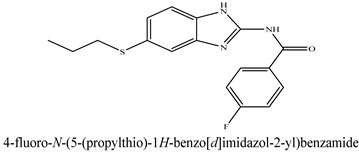	12.14 ± 0.32	8.16 ± 0.51
8	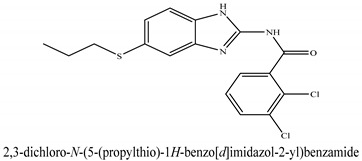	24.23 ± 0.28	14.94 ± 0.31
9	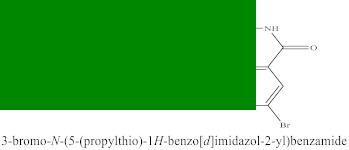	13.05 ± 0.29	8.41 ± 0.18
10	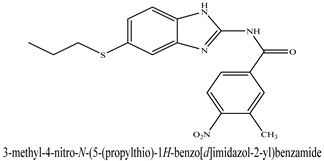	8.399 ± 0.23	4.73 ± 0.37
11	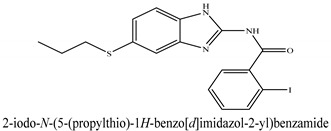	11.93 ± 0.61	14.32 ± 0.37
12	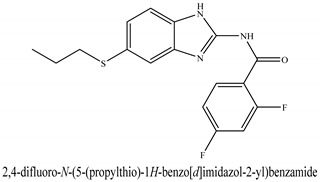	11.79 ± 36	19.22 ± 0.17
13	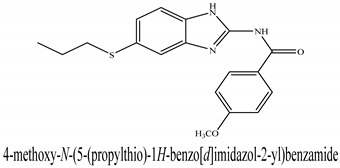	11 ± 0.81	15.12 ± 0.09
14	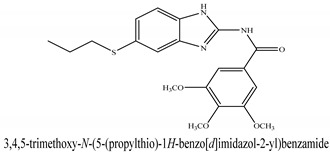	8.691 ± 0.83	5.51 ± 0.65
15	‘ 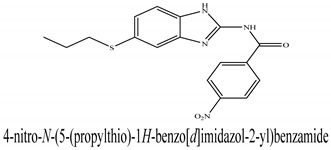	14.77 ± 0.32	10 ± 0.41
16	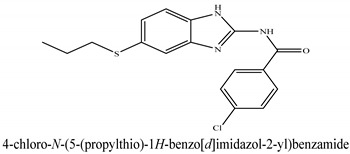	12.93 ± 0.21	16.7 ± 0.36
17	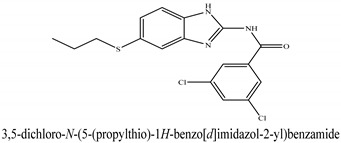	16.15 ± 0.26	9.6 ± 0.19
18	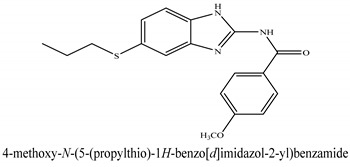	9.04 ± 0.51	7.3 ± 0.07

**Table 2:** Primer designs.

Genes	Primer Sequence	Products Sizes
albumin (F)	GCTGTAGTGGATCCCTGGTG	196
albumin (R)	GCTGTAGCCTTGGGCTTG	
CK-18 (F)	TGAGACAGAACTAGCCATGC	208
CK-18 (R)	CACTTCCACAGTCAATCCAG	
CK-8 (F)	CTCACTAGCCCTGGCTTCAG	232
CK-8 (R)	ACAGCTGTCTCCCCGTGA	
*BAX* (F)	TGGAGATGAACTGGACAGCA	152
*BAX* (R)	CAAAGTAGAAGAGGGCAACCAC	
TNF-α (F)	ACGGCATGGATCTCAAAGAC	162
TNF-α (R)	GGAGGTTGACTTTCTCCTGGTA	
caspase-3 (F)	TGTCATCTCGCTCTGGTACG	220
caspase-3 (R)	AAATGACCCCTTCATCACCA	
NF-κβ (F)	GCACCTGTTCCAAAGAGCAC	200
NF-κβ (R)	GTGGAGTGAGACATGGACACAC	
Bcl-xL (F)	TTCGGGATGGAGTAAACTGG	150
Bcl-xL (R)	AAGGCTCTAGGTGGTCATTCAG	
*BCl_2_*(F)	GATGACTTCTCTCGTCGCTAC	182
*BCl_2_*(R)	ACGCTCTCCACACACATGAC	
β-actin (F)	ACTGCTCTGGCTCCTAGCAC	115
β-actin (R)	ACATCTGCTGGAAGGTGGAC	

## Data Availability

Data are contained within the article and Supplementary Materials.

## Supplementary Materials

The following supporting information can be downloaded at: https://www.mdpi.com/article/10.3390/ph16020306/s1, **Figure S1.** Morphology of rat hepatocytes cells in in vitro culture isolated from male Wister rat: (**A**) After 2 h of culturing, the cultured cells were spherical in shape and were bi-nucleated in phase-contrast microscope. **(B**) After 24 h of culturing, hepatocytes were polygonal in shape and were bi-nucleated, showing the morphology of normal hepatocyte (20×; scale bar: 100µm). **Figure S2.** Characterization of cultured hepatocytes: (**A**) Real-time PCR analysis for the expression of hepatic marker (albumin, CK-8 and CK-18) in cultured cells. β-actin was used as endogenous control. (**B**) PAS-staining for stored glycogen in cultured hepatocytes. The presence of glycogen is shown by purple color. What is the function of normal hepatocytes? (**C**) Immunostaining of cultured hepatocytes for the hepatic marker expression at the protein level. DAPI (blue) was used to stain nuclei. Cultured hepatocytes cells were positive for (**a**) albumin, (**b**) CK-8, (**c**) CK-18 and (**d**) AFP (10×; scale bar: 100 µm). **Figure S3.** In vitro Hepatocytes injury culture: (**A)** LDH assay of normal and CCl_4_ (5 mM) injured hepatocytes. (**B**) trypan blue assay for normal hepatocytes and CCl_4_ (5 mM) injured hepatocytes. (**C**) Glutathione assay for normal and CCl_4_ (5 mM)-injured hepatocyte; (**D**) Real-time PCR analysis of in vitro CCI_4_ (5 mM) injury culture of hepatocytes. **Figure S4.** in vitro screening of compounds: (**A**) LDH assay of 18-compound benzimidazol derivatives series. (**B**) trypan blue assay of 18-compound benzimidazol derivatives series. (**C**) Glutathione (GSH) assay of 18-compound benzimidazol derivatives series.
