# Supplementary material for: Combinatorial Therapeutic Potential of Stem Cells and Benzimidazol Derivatives for the Reduction of Liver Fibrosis
Source: Pharmaceuticals (Basel). 2023 Feb 15;16(2):306. doi: 10.3390/ph16020306 (PMC9965641; doi:10.3390/ph16020306)
Supplement: Supplementary file 1 [file pharmaceuticals-16-00306-s001.zip › pharmaceuticals-2196343-supplementary.pdf]

Combinatorial Therapeutic Potential of Stem Cells and Benzimidazol Derivatives for the Reduction of Liver Fibrosis

Supplementary Data:

Figure S1. Morphology of rat hepatocytes cells in *In Vitro* culture isolated from male Wister rat. (**A**) After 2 hrs of culturing, the cultured cells were spherical shape and were bi-nucleated in phase contrast microscope; **(B**) After 24hrs of culture hepatocytes were polygonal in shape and were bi-nucleated. Showing the morphology of normal hepatocyte (20X, scale bar 100μm).

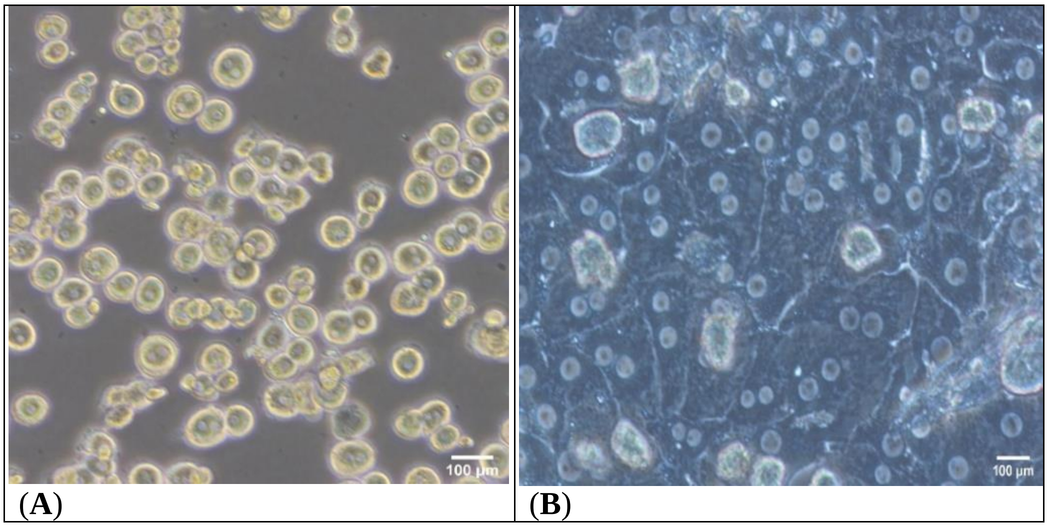

(**A**) (**B**)

Figure S2. Characterisation of Cultured Hepatocytes.(**A**) Real time PCR analysis for expression of hepatic Marker **(***Albumin*,*CK-8* and *CK-18*) in cultured cell s. β-actin was used as endogenous control. (**B**) PAS staining for stored glycogen in cultured hepatocytes; The presence of glycogen are shown by Purple color. This is the function of normal.

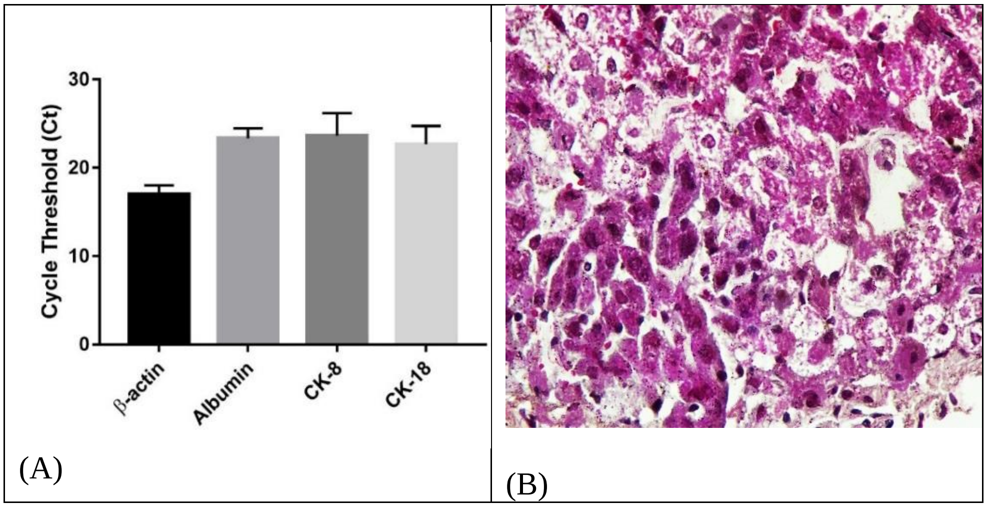

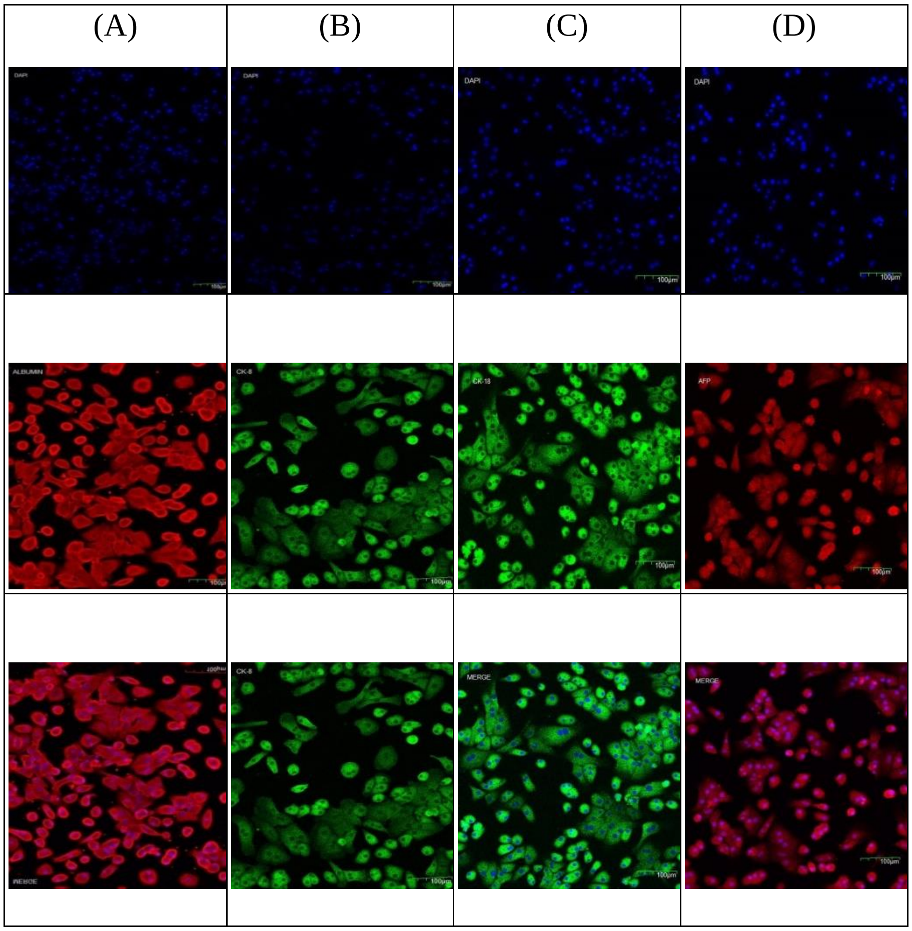

Figure S3. *In vitro* Hepatocytes injury Model. (**A)** LDH assay of normal and CCl_4 (5mM) injured hepatocytes. (**B**) Trypan blue assay for normal hepatocytes and CCl_4 (5mM) injured hepatocytes. (**C**) Glutathione assay for normal and CCl_4 (5mM) injured hepatocyte. (**D**) Real time PCR analysis of *In Vitro* CCI4 (5mM) Injury Model of Hepatocytes.

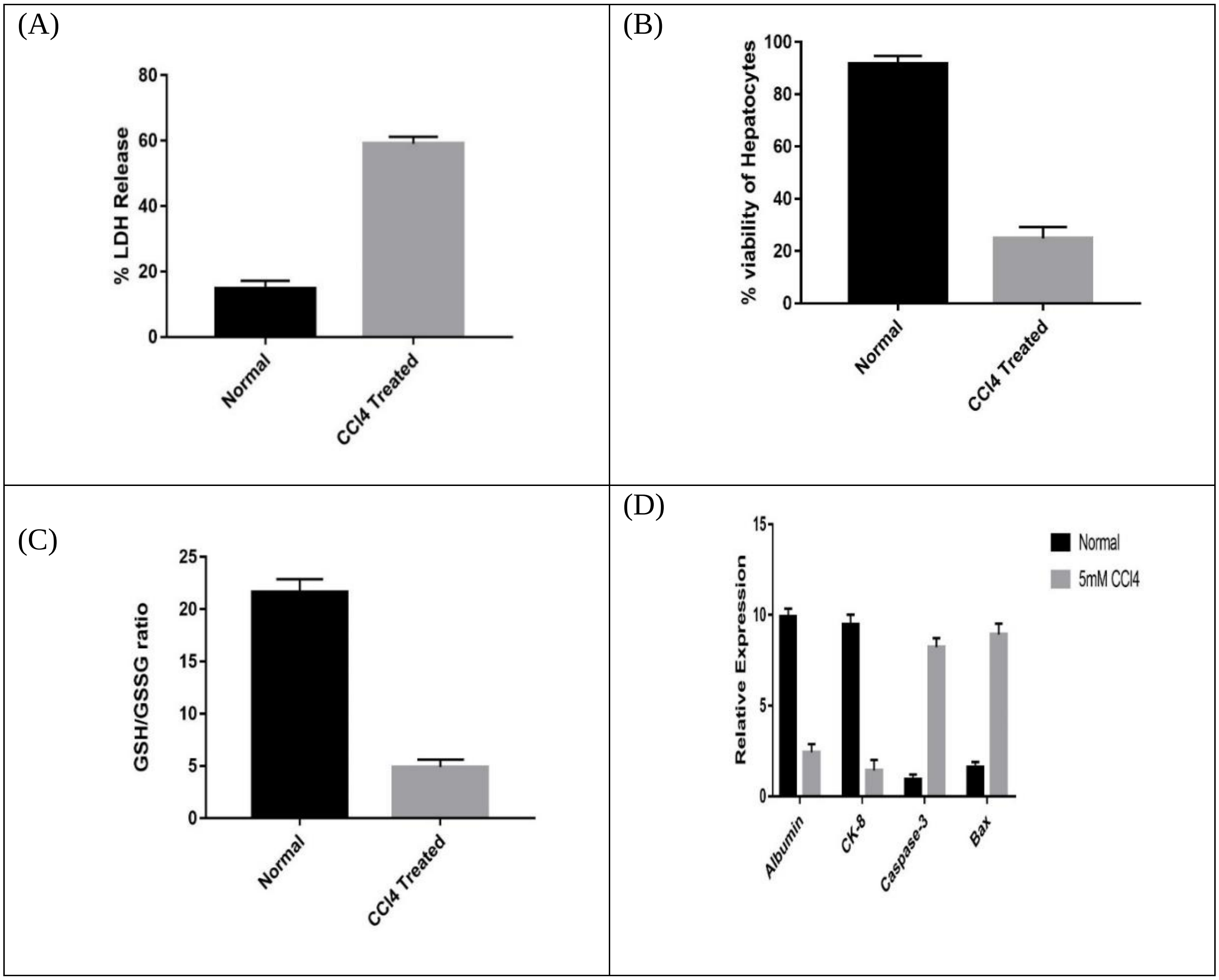

Figure S4. *In Vitro* Screening of Compounds. (**A**) LDH assay of 18 compounds series Benzimidazol derivatives. (**B**) Trypan blue assay of 18 compounds series Benzimidazol derivatives. (**C**) Glutathione (GSH) assay of 18 compounds series Benzimidazol derivatives.

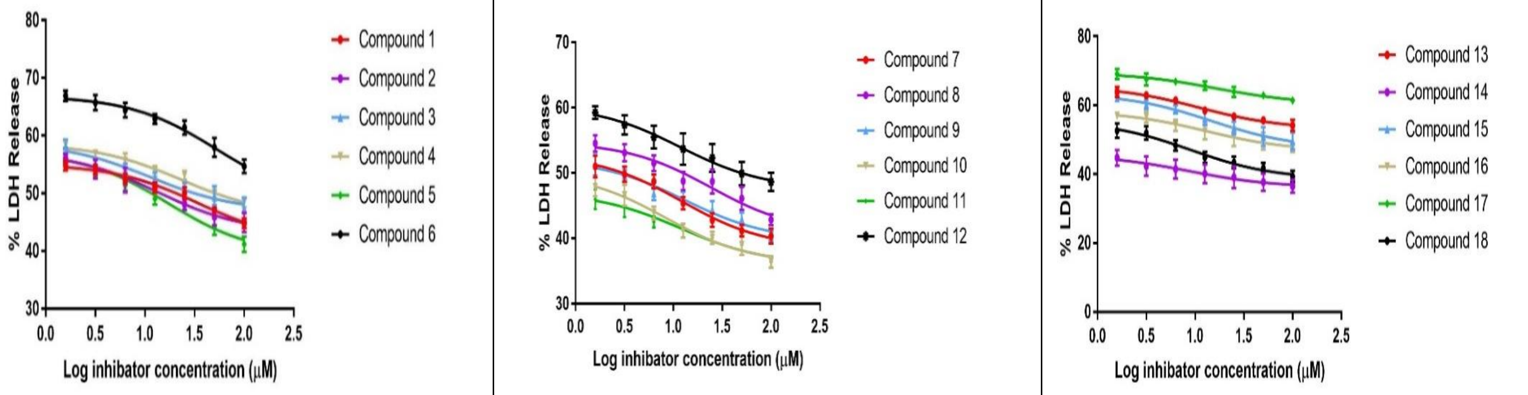

(A)

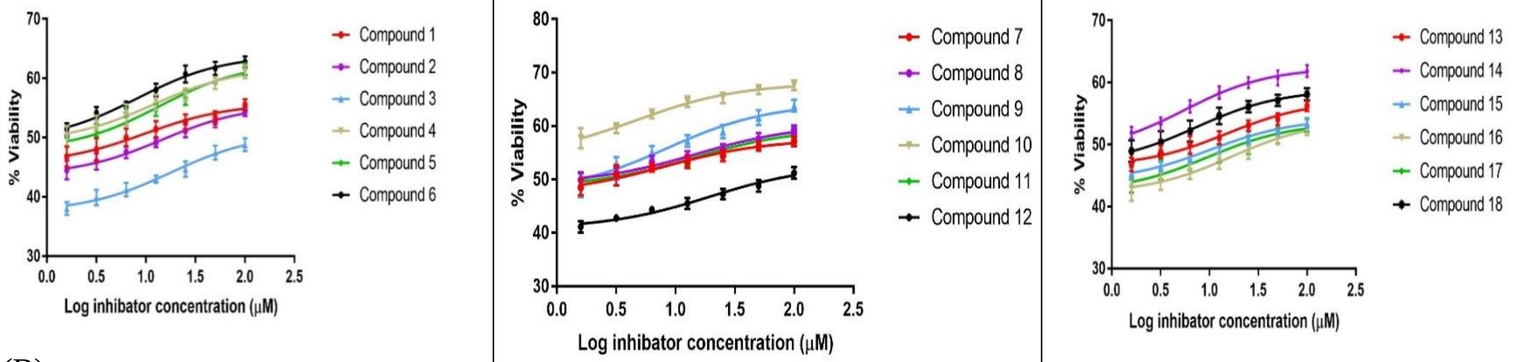

(B)

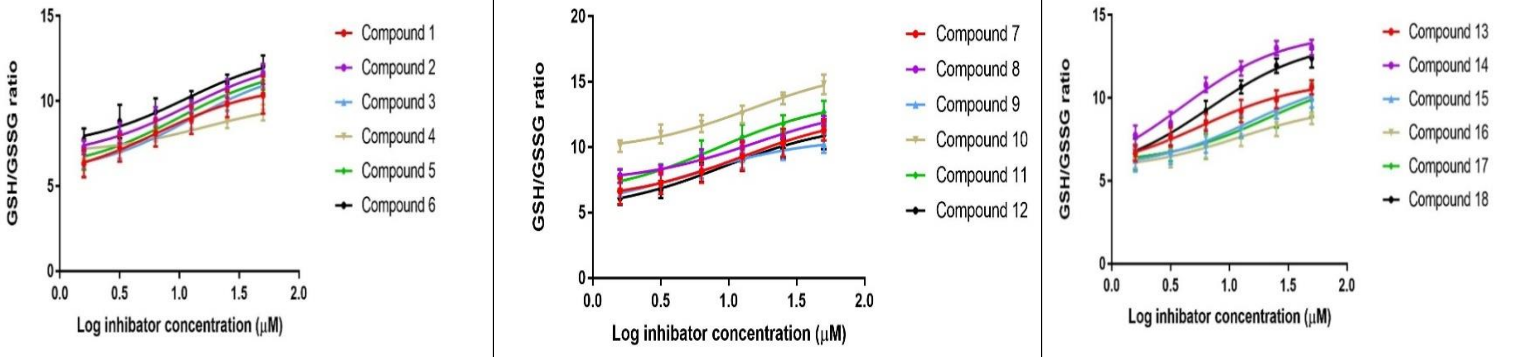

(C)
